# Supplementary figures and images for: Study on underwater noise characteristics and mechanism of discharge flow from wide-crested weir
Source: PLoS One. 2026 Mar 20;21(3):e0332839. doi: 10.1371/journal.pone.0332839 (PMC13004519; doi:10.1371/journal.pone.0332839)

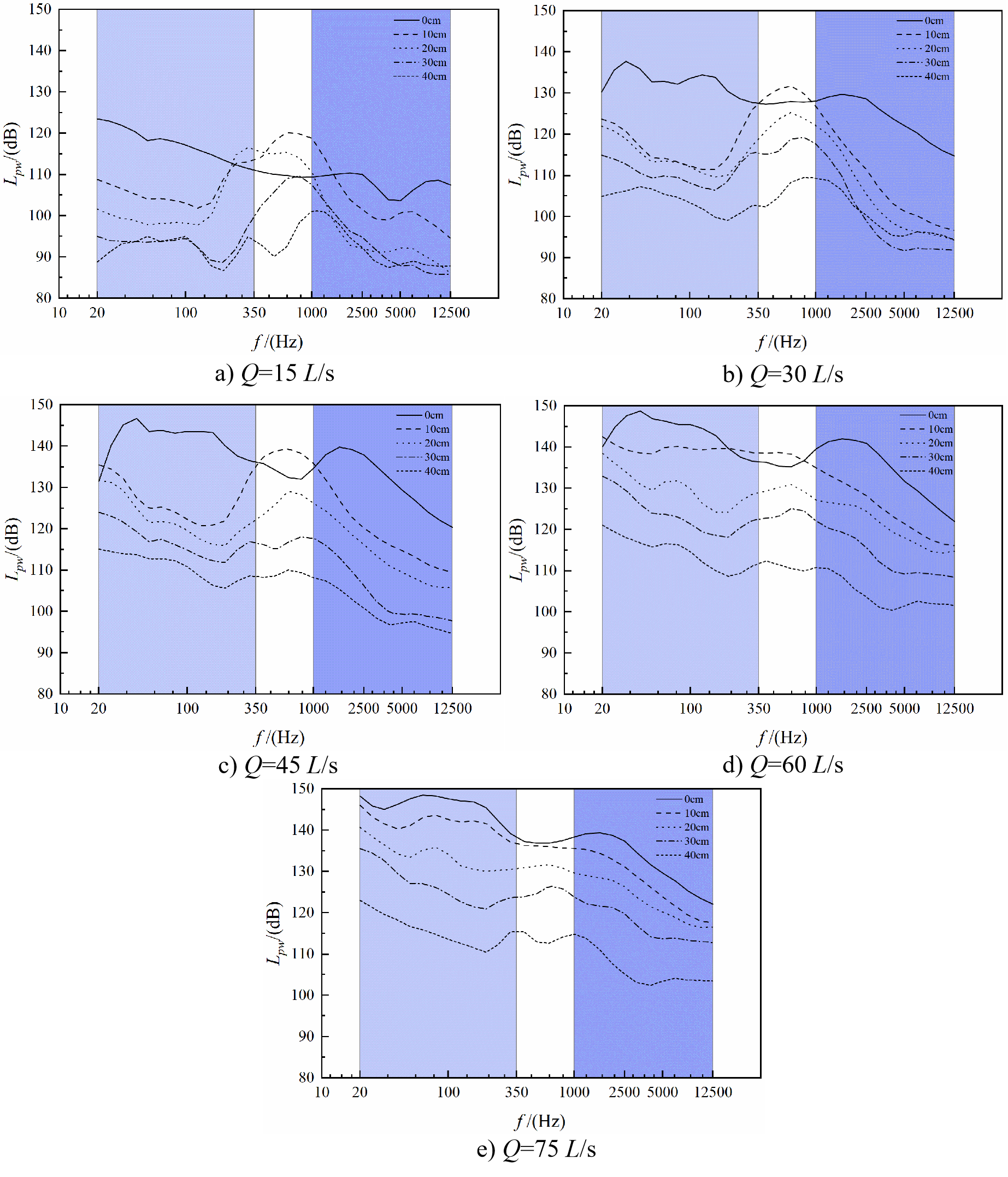

Supplement: S1 Fig — (TIF) [file pone.0332839.s001.tif]

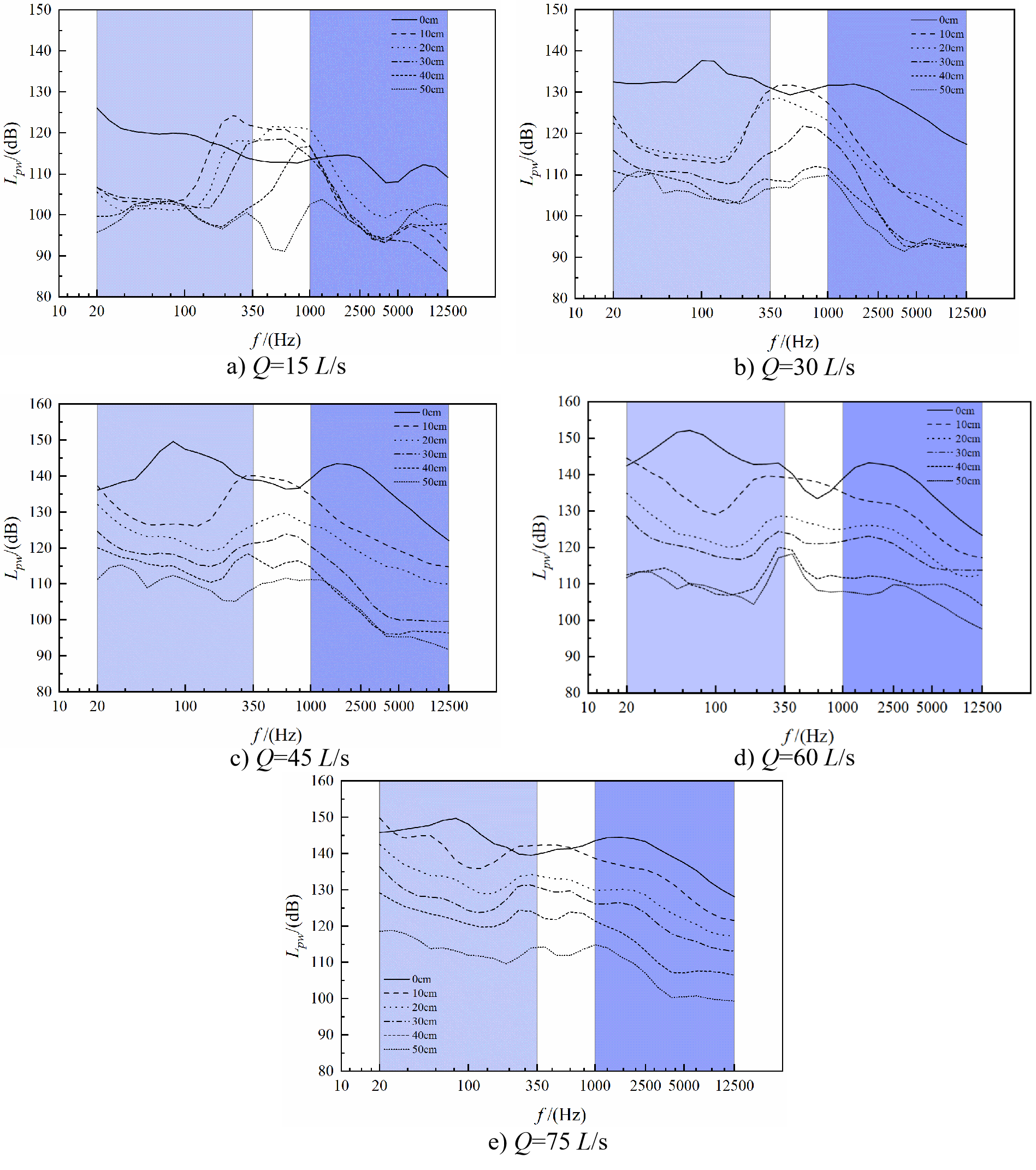

Supplement: S2 Fig — (TIF) [file pone.0332839.s002.tif]

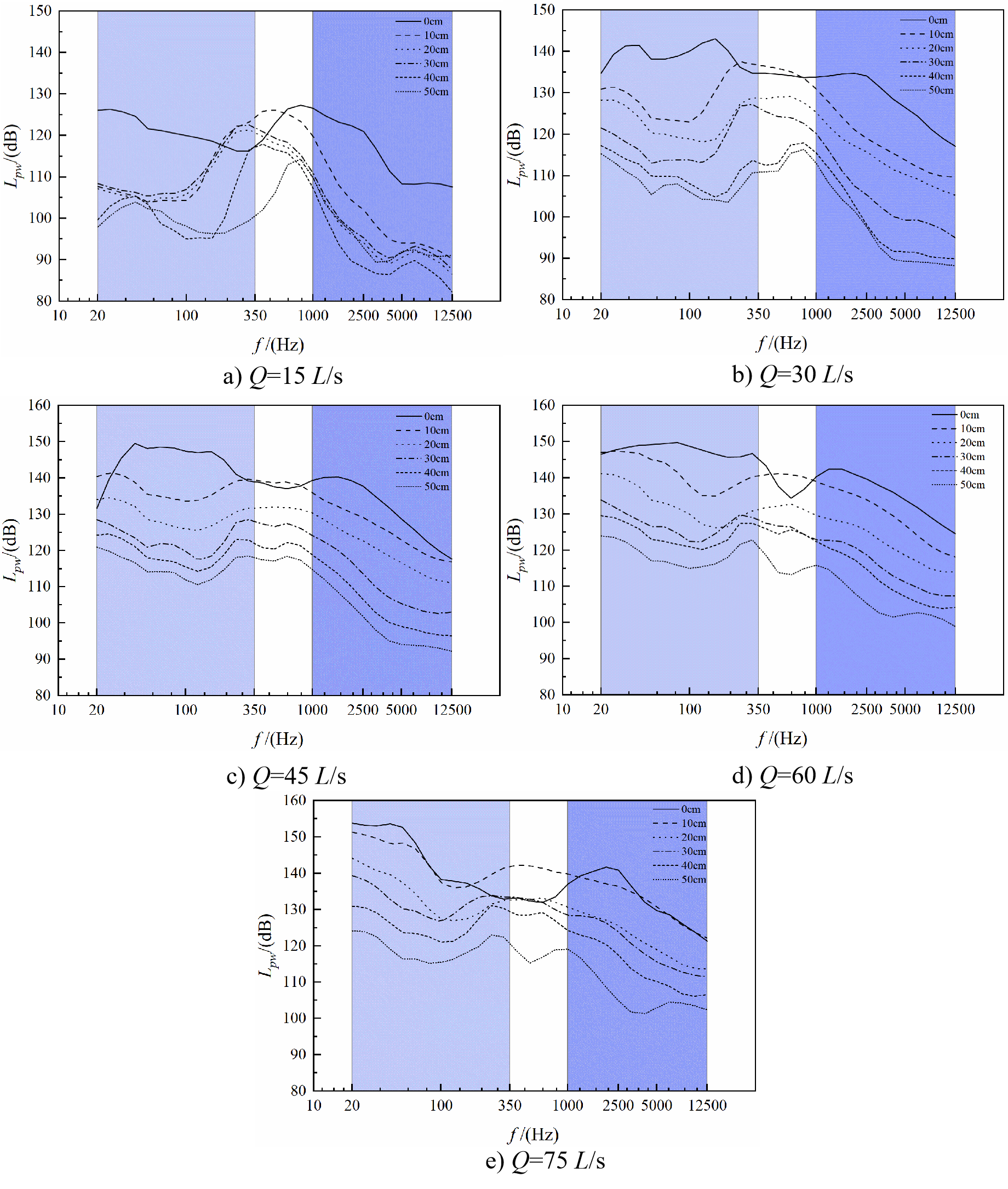

Supplement: S3 Fig — (TIF) [file pone.0332839.s003.tif]

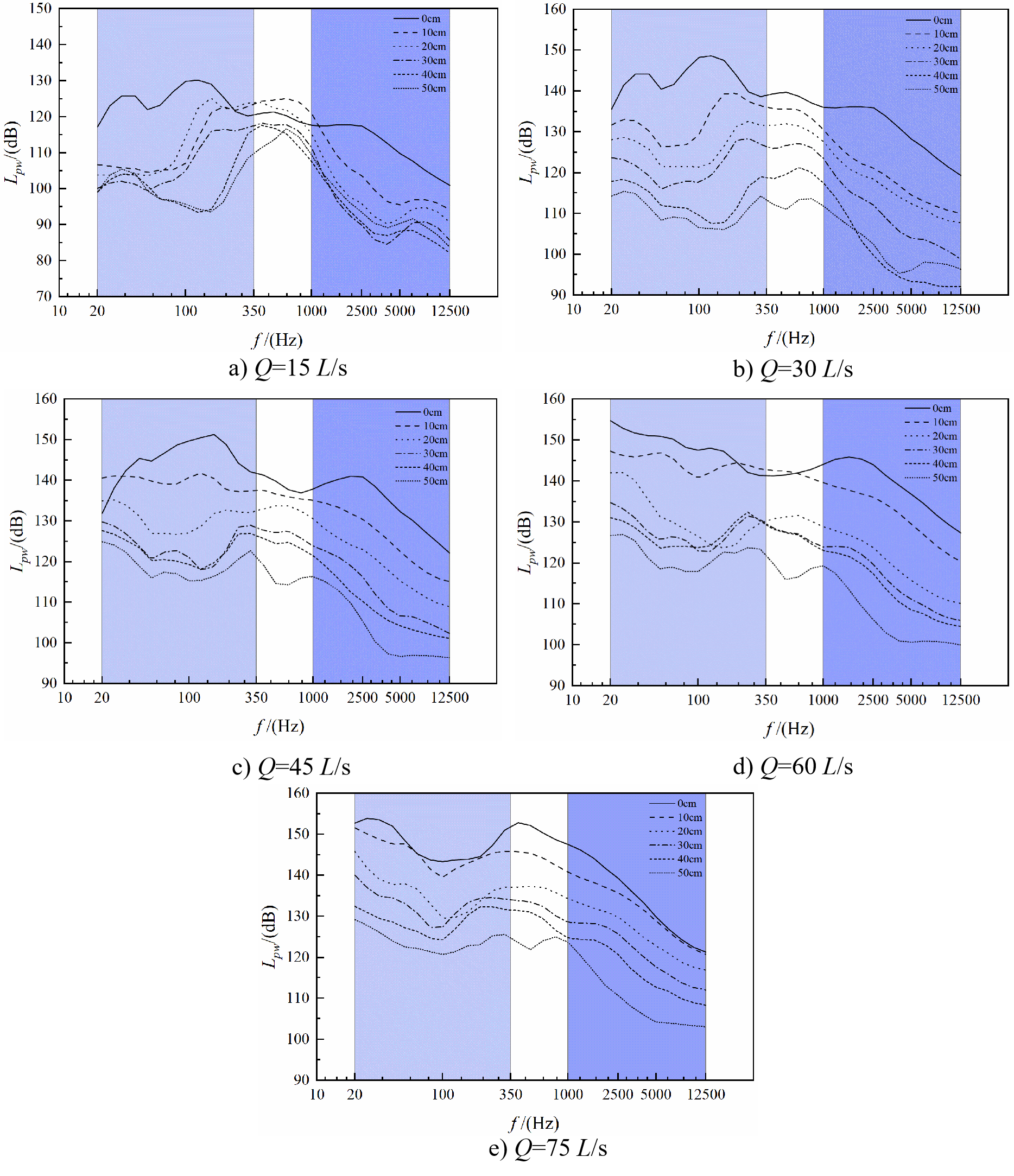

Supplement: S4 Fig — (TIF) [file pone.0332839.s004.tif]

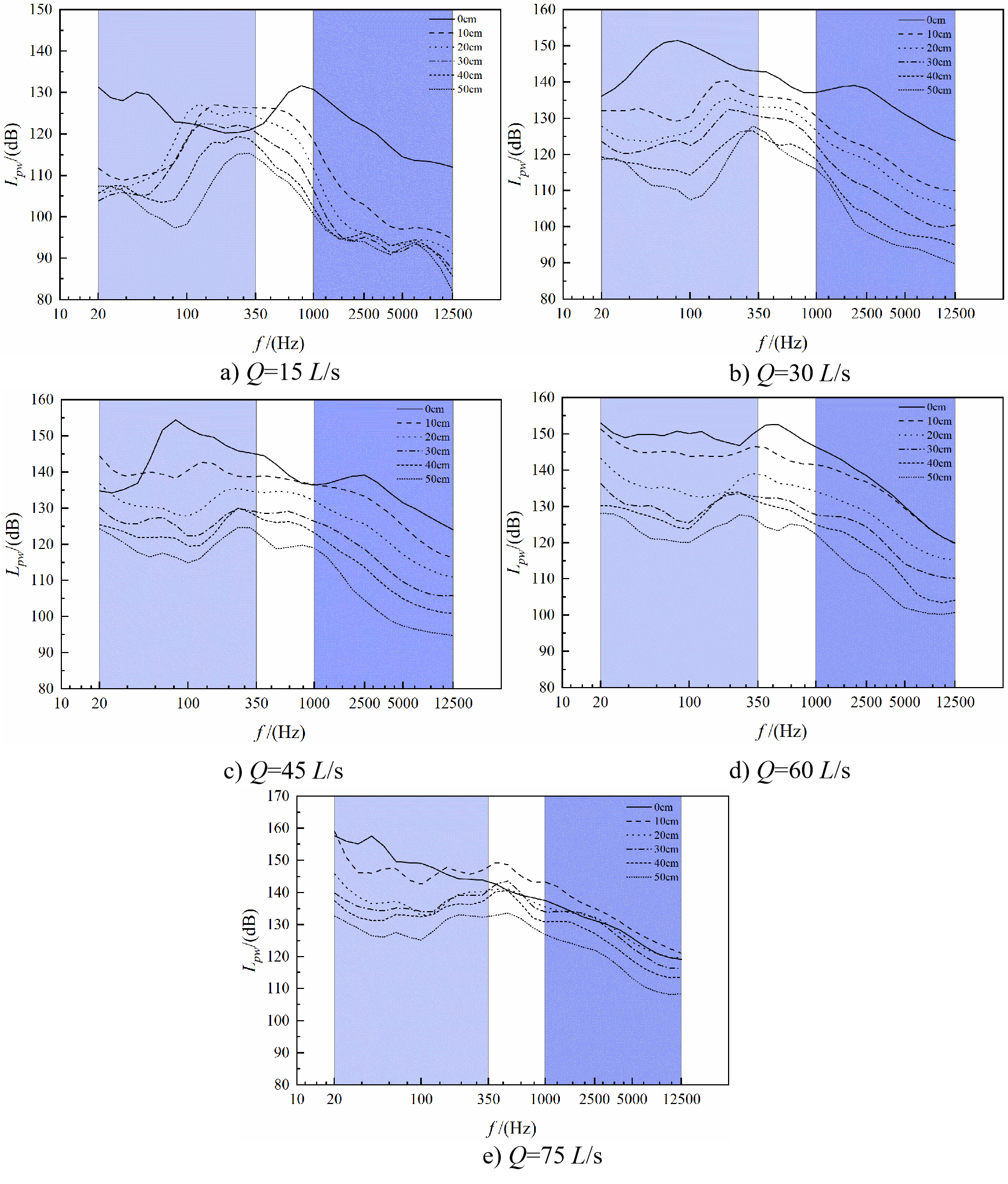

Supplement: S5 Fig — (TIF) [file pone.0332839.s005.tif]
