# Supplementary material for: Study on underwater noise characteristics and mechanism of discharge flow from wide-crested weir
Source: PLoS One. 2026 Mar 20;21(3):e0332839. doi: 10.1371/journal.pone.0332839 (PMC13004519; doi:10.1371/journal.pone.0332839)
Supplement: Attachment — Submitted filename: ReviewPLOS2025.pdf [file pone.0332839.s006.pdf]

Review of the article entitled "Study on underwater noise characteristics and mechanism of discharge flow from wide-crested weir" by Q. Shui, D. Wang, Y. Chen, T. Yu, Y. Fan, and Y. Dai.

This works integrates physical experiments and numerical simulations to provide theoretical guidance and technical support for mitigating underwater noise generated by discharge flow from wide-crested weirs. Although the initial intent of the authors seems valuable, the reviewer has detected flaws in the presentation and analysis of the experimental results as well as in the grid convergence process impacting numerical results that prevents its publication in PLOS ONE in the present form.

To detail the issue, the reviewer starts with the presentation of the experimental results. First, it is not clear why only two measures are available for each configuration. The authors have spent a lot of time and efforts to the detailed design and operation of an experimental test-bench devoted to the acoustic analysis of the flow generated by a wide-crested weir. They have gathered data for different volume flow rates (Q), different downstream water heights (h) and weir heights (H). Yet the only available measure is the pressure signal from a sigle pressure probe located 30cm downstream of the weir. This appears somewhat puzzling to the reviewer. Two pressure quantities are analyzed which represent the average spectral intensity L_{pw} and the rms intensity of the signal (Fluctuating Pressure SPL).
That said, the presentation of the experimental results is not satisfactory at all. Fig 6 is not clear because of the perspective effect which prevents the reader from determining the precise level of L_{pw} as a function of H, Q or h. Curves should be used on graphs and if the amount of information is too large, an appendix should be created. The exact same comment can be made for Fig 7. Pearson correlation factors between L_{pw}, SPL, Q, H and h are interesting yet quite expected. The authors should consider determining the R-2 factor to ensure the causality relation between the evolutions of the different variables. The analysis of the frequency content of the pressure spectra (Figs 9, 10, 11) is again not satisfactory. One cannot directly observe the values of the frequency peaks mentioned by the authors. Again, appendices should be used for the presentation of extra data. Given these comments, the experimental results do not represent a firm base to compare the numerical simulations to, which goes against the authors original intent.

Regarding the numerical simulations, one could already question the numerical choices made by the authors which are quite dissipative (URANS, second order fluxes and $k - \epsilon$ turbulence model). For better accuracy, the authors should consider two-phase flow LES, with more advanced turbulence models (such as dynamic smagorinsky model), higher order schemes for the convective fluxes and most importantly a better tracking method for the interface (such as combined VOF/levelset). Without these numerical ingredients, only rough trends can be recovered at best and there is no hope the turbulent acoustic spectra can be numerically recovered.
Yet this is not the main criticism the reviewer has. The grid convergence analysis is not satisfactory which definitely questions the numerical results. The authors have access to the SPL for each case so why this quantity is not used to analyze the numerical convergence? Indeed one could expect that the mesh size should be determined with respect to an acoustic information and not barely the mean value of the pressure at the location of the microphone in the experiment.
Even if the first remark about numerical accuracy can probably be set aside because of the computing hours accessibility, a proper grid convergence analysis using the same data analyzed in the core of the article is really expected for studies to be published in journal PLOS ONE.

Given those two points, the reviewer rejects the publication of this study in its present form. The original intents of the authors are interesting though and they should (a) provide more experimental results, (b) present them in a more understandable way and (c) assess the numerical grid convergence more properly. Given these changes they should resubmit the paper as an original contribution.
