# Supplementary material for: Study on underwater noise characteristics and mechanism of discharge flow from wide-crested weir
Source: PLoS One. 2026 Mar 20;21(3):e0332839. doi: 10.1371/journal.pone.0332839 (PMC13004519; doi:10.1371/journal.pone.0332839)
Supplement: Attachment — Submitted filename: Review-01.docx [file pone.0332839.s007.docx]

This manuscript is about underwater noise characteristics and mechanism of discharge flow from wide-crested weir and it provides theoretical guidance and technical support for mitigating underwater noise generated by discharge flow from wide-crested weirs. The manuscript topic is within the scope of the journal. The manuscript is well-written and provides a comprehensive study for the noise generated by weir discharging. The following comments are provided:

Comment #1: (Pages: , Line: )

Please, insert the pages numbers so that it will be easy for reference the comment’s locations.

--------------------------------------------------------------------------------------------------------------------

Comment #2: (Page: 8, Line: 48)

The authors mentioned that Guo et al. [21, 22] established relationships between discharge noise, weir type, and flow rate through spectral and fluctuating-pressure analyses. It seems that the current study is similar to Guo’s studies, so explain and highlight the difference/differences between your study and their study and compare your results with their results.

--------------------------------------------------------------------------------------------------------------------

Comment #3: (Page: 9, Line: 67)

For the text “and fluctuating pressure SPL”, why the repetition of the SPL? I think it should be deleted.

--------------------------------------------------------------------------------------------------------------------

Comment #4: (Page: 13, Line: 164-184)

Is it a 2D problem? Is it a steady state problem?

--------------------------------------------------------------------------------------------------------------------

Comment #5: (Pages: 13, Line: 164-182)

How did the boundary conditions at the inlet obtained for the numerical simulation? Please clarify that.

--------------------------------------------------------------------------------------------------------------------

Comment #6: (Pages: 14, Line: 203)

Where is Fig. 3.1? Only Fig.3 is detected.

--------------------------------------------------------------------------------------------------------------------

Comment #7: (Page: 19, Line: 280)

What is the device used to obtained the data in Fig. 9?

--------------------------------------------------------------------------------------------------------------------

Comment #8: (Page: 23, Line: 360)

What is the method/device used to obtain the data in Fig.12?

--------------------------------------------------------------------------------------------------------------------

Comment #9: (Page: 25, Line: 389)

The x and y labels in Fig. 13 is not clear. It is “x/m” is this a dimensionless? I think you mean x (m), “x” is the coordinate direction and “m” is the unit. If this is what you mean, please convert the label to x (m), y (m) and do the same for all figures after Fig. 13.

--------------------------------------------------------------------------------------------------------------------

Comment #10: (Page: 26-36, Line: 406-602)

The results of the figures (14-26) shown are based on numerical simulations for unsteady problem (at the beginning it is unsteady but it will reach the steady state at the convergence) whereas the real problem is unsteady state because there is a fluctuating in the pressure and noise. Could you explain that? I mean you are using steady result to explain an unsteady problem.

--------------------------------------------------------------------------------------------------------------------

Comment #11: (Page: 39, Line: 650)

Since that it is clear from Fig. 28 the relation between the H and noise, I think that Fig.29 can be deleted.

--------------------------------------------------------------------------------------------------------------------

Comment #12: (Page: 39, Line: 652)

When using the abbreviation RMS it should be identify for what quantity it measured. For example, Pressure RMS. RMS is a general quantity such as average, so it should defined by relating it to the quantity it used to measure. In this study, it referred to pressure.

--------------------------------------------------------------------------------------------------------------------

Comment #13: (Page: 25-32 & 32-36, Line: 388-507 & 508-603)

In the manuscript there are too many figures that I think they can be reduced because they have the same meaning. For example, the data in Figs 13-22 are based on the kinetic energy which is based on velocities quantities. Also, the Figs 23-26 are based on vortex structure which are also depend on velocities quantities so the results of increasing or decreasing tends will be the similar for that in Figs. 13-22.

--------------------------------------------------------------------------------------------------------------------
