# Supplementary material for: Study on underwater noise characteristics and mechanism of discharge flow from wide-crested weir
Source: PLoS One. 2026 Mar 20;21(3):e0332839. doi: 10.1371/journal.pone.0332839 (PMC13004519; doi:10.1371/journal.pone.0332839)
Supplement: Attachment — Submitted filename: Response to Reviewers.docx [file pone.0332839.s008.docx]

Dear Editors and Reviewers:

Thank you for your letter and for the reviewers’ comments concerning our manuscript entitled “***Study on underwater noise characteristics and mechanism of discharge flow from wide-crested weir***” (ID: PONE-D-25-48325). Those comments are all valuable and very helpful for revising and improving our paper, as well as the important guiding significance to our researches. We have studied comments carefully and have made correction which we hope meet with approval. ***Revised portion are marked red in the Marked Revised Manuscript.*** The main corrections in the paper and the responses to the reviewer’ comments are as following:

**Reviewer #1:**

1. **Comment**: “Please, insert the pages numbers so that it will be easy for reference the comment’s locations.”

**Response**: *Thank you for your comment. We have added page numbers and setted line number for each page in the Revised Manuscript.*

2. **Comment**: “(Page: 8, Line: 48)The authors mentioned that Guo et al. [21, 22] established relationships between discharge noise, weir type, and flow rate through spectral and fluctuating-pressure analyses. It seems that the current study is similar to Guo’s studies, so explain and highlight the difference/differences between your study and their study and compare your results with their results.”

**Response**: *Thank you for your comment. In contrast to the trend-based conclusions regarding various factors derived by Guo et al.*[1-2] *through physical experiments, this study employs a hybrid approach combining physical experimentation and numerical simulation. This methodology enables the precise capture of microscopic flow field details, such as gas-liquid distribution and vorticity evolution, revealing that vortex breakup and bubble oscillation are the core sources of underwater noise. Furthermore, this paper establishes a quantitative coupling relationship between fluctuating pressure and noise, identifying the downstream static water depth (h) as the primary factor in noise suppression. These findings provide direct theoretical support and a quantitative basis for the design of noise mitigation measures in engineering.*

***Reference:***

[1] Guo W D, JIN X, ZHAO G Y, et al. Experimental study on discharge noise of WES Weir under different conditions [J]. Yellow River. 2017, 39(02): 116-119.
[2] Guo WD, Meng F, Jin MX, et al. Study on fluctuating pressure of discharge flow from rubber dam and WES Weir. Yellow River. 2017, 39(5): 97-103, 108.
3. **Comment**: “(Page: 9, Line: 67) For the text “and fluctuating pressure SPL”, why the repetition of the SPL? I think it should be deleted.”

**Response**: *Thank you for your comment and we sincerely thank the reviewer for careful reading. Based on data derived from physical experiments in our original manuscript, correlation analysis is conducted to quantify the effects of inflow flow, downstream hydrostatic depth and wide-crested weir height on the underwater noise sound pressure level and fluctuating pressure sound pressure level. Furthermore, these effects are analyzed by integrating results from numerical simulation. The underwater noise sound pressure level and fluctuating pressure sound pressure level serve as the principal metrics of our research and are abbreviated as “underwater noise sound pressure level (SPL) and fluctuating pressure SPL”.*

4. **Comment**: “(Page: 13, Line: 164-184) Is it a 2D problem? Is it a steady state problem?”

**Response**: *Thank you for your comment. Analysis of the time-series pressure from physical model tests reveals the unsteady nature of the flow over the broad-crested weir. In alignment with the experimental setup, the numerical investigation employs a three-dimensional computational domain. Therefore, this study addresses three-dimensional unsteady flow dynamics. In order to help readers quickly understand the inlet boundary condition of this paper, the necessary explanations have been incorporated into the Revised Manuscript as follows:*

***(on Page 8, Lines 5-6)*** *The standard k–ε turbulence model is applied to simulate three-dimension unsteady incompressible flow without heat conduction.*

***(on Page 11, Line 1)*** *The no-slip condition is used in the spanwise direction.*

5. **Comment**: “(Pages: 13, Line: 164-182)How did the boundary conditions at the inlet obtained for the numerical simulation? Please clarify that. ”

**Response**: *Thank you for your comment. The governing equations were simulated using the open-source Computational Fluid Dynamics software OpenFOAM v7. Inlet uses the boundary condition variableHeightFlowRateInletVelocity (https://cpp.openfoam.org/v7/classFoam_1_1 variableHeightFlowRateInletVelocityFvPatchVectorField.html). The boundary condition calculates an averaged velocity (avgU) by dividing the desired flow rate (q in our Manuscript) by the current cross-sectional area of the water phase (*$\alpha$*). The avgU is given as*

$avgU=\frac{q}{\int_{S} \alpha dA}$ *(1)*

*The avgU is then applied across the water phase on the boundary path. As shown in Eq.(1), a uniform velocity across boundaries of cells with* $\alpha=1.0$*, zero velocity across boundaries of cells with* $\alpha=0.0$*, and an intermediate velocity for cells containing the air-water interface. Consequently, our requirement of inlet boundary condition (* *(Page: 7, Line: 14)* The water and gas boundary conditions are automatically adjusted based on the water depth.*) that sets the discharge is met. In order to help readers quickly understand the inlet boundary condition of this paper, the necessary explanations have been incorporated into the Revised Manuscript* ***(on Page 10, Lines 17-18 in the Revised Manuscript)****.*

6. **Comment**: “(Pages: 14, Line: 203)Where is Fig. 3.1? Only Fig.3 is detected. ”

**Response**: *Thank you for the reminder. We are very sorry for our incorrect writing in this part. We have corrected this part in the Revised Manuscript (****on Page 12, Line 18)****.*

7. **Comment**: “(Page: 19, Line: 280)What is the device used to obtained the data in Fig. 9?”

**Response**: *Thank you for your comment. In Fig.9* ***(on Pages 21-22 in the Revised Manuscript)****, the left figure at each condition shows the time–frequency characteristics obtained from the wavelet analysis, and the right part is obtained from the Fast Fourier transform (FFT). In Figs.10-11, the same method is used.*

8. **Comment**: “(Page: 23, Line: 360)What is the method/device used to obtain the data in Fig.12?”

**Response**: *Thank you for your comment. Fig.12 is obtained by analyzing the numerical simulation results using the post-processing soft Tecplot.*

9. **Comment**: “(Page: 25, Line: 389)The x and y labels in Fig. 13 is not clear. It is “x/m” is this a dimensionless? I think you mean x (m), “x” is the coordinate direction and “m” is the unit. If this is what you mean, please convert the label to x (m), y (m) and do the same for all figures after Fig. 13.”

**Response**: *Thank you for your comment. We have converted all the same labels in the revised manuscript.*

10. **Comment**: “(Page: 26-36, Line: 406-602)The results of the figures (14-26) shown are based on numerical simulations for unsteady problem (at the beginning it is unsteady but it will reach the steady state at the convergence) whereas the real problem is unsteady state because there is a fluctuating in the pressure and noise. Could you explain that? I mean you are using steady result to explain an unsteady problem.”

**Response**: *Thank you for your comment, and we apologize if the description of the numerical method was not sufficiently clear. We wish to clarify that the numerical simulations conducted in this study were indeed unsteady simulation, not steady-state simulations. We utilized the standard* $k-\varepsilon$ *turbulence model combined with the VOF method using the interFoam solver in OpenFOAM, which is specifically designed for transient multiphase flow problems. The specific reasons supporting this are as follows:*

1) *As shown in the Governing Equations (5)-(6)* ***(on Page 8, Lines 8-9 in the Revised Manuscript)****, the momentum equations include time-dependent terms (*$\frac{\partial\overline{U}_{i}}{\partial t}$*), explicitly indicating that the model solver for time-varying flow fields.*
2) *As described* ***on Page 10, Lines 6-13 in the Revised Manuscript****, we employed the PISO (Pressure-Implicit with Splitting of Operators) algorithm for pressure-velocity coupling and used a second-order implicit backward Euler method for time integration. These are standard methods for unsteady flow simulations.*
3) *The convergence or steady state mentioned in the context of our simulation refers to the statistically stationary state. In our simulation process (total time = 60 s according to the experimental sampling time on* ***Page 6, Line 5 in the Revised Manuscript****), the flow evolves from the initial conditions until the mean flow quantities. Once this statistically stationary stage is reached, the instantaneous flow variables continue to fluctuate over time due to turbulence and phase interaction* ***(on Page 27, Fig 13 in the Revised Manuscript)****. It is precisely these time-domain fluctuations that we extracted to calculate the fluctuating pressure and SPL.*

*Therefore, Figures 14-26 do not represent results from a static, steady-state solution. Instead, they present either instantaneous snapshots of the dynamic flow field or statistical analyses (such as RMS values and frequency spectra) derived from the times-series data collected during the statistically stationary fluctuating stage.*

11. **Comment**: “(Page: 39, Line: 650)Since that it is clear from Fig. 28 the relation between the H and noise, I think that Fig.29 can be deleted.”

**Response**: *Thank you for your comment to streamline the Manuscript. However, we respectfully believe that Fig. 29 cannot be removed, as it provides information that is not fully represented in Fig. 28. Fig. 28 illustrates the relationship between incoming flow rate (Q) and fluctuating pressure SPL under various weir heights (H) and downstream static water depths (h), where the H serves as a parametric condition rather than the primary controlling variable. In contrast, Fig. 29 explicitly investigates the effect of H on fluctuating pressure SPL under different Q and h, with the H treated as the independent variable. Therefore, Fig. 29 is essential for isolating and quantifying the influence of H itself, which cannot be clearly distinguished from Fig. 28 alone. In combination with Fig. 27, these three figures address different analytical perspectives and together provide a more complete understanding of the combined and individual effects of Q, h, and H on fluctuating pressure SPL. For this reason, we believe that Fig. 29 is complementary rather than redundant and should be retained.*

12. **Comment**: “(Page: 39, Line: 652)When using the abbreviation RMS it should be identify for what quantity it measured. For example, Pressure RMS. RMS is a general quantity such as average, so it should defined by relating it to the quantity it used to measure. In this study, it referred to pressure. ”

**Response**: *Thank you for your helpful comment. We agree that RMS is a general statistical quantity and should be associated with the physical variable being measured. In this study, RMS refers specifically to the root mean square of fluctuating pressure. To eliminate any ambiguity, we have revised RMS as fluctuating pressure RMS throughout the Revised Manuscript. The corresponding definition is provided* ***on Page 7, Line 17 in the Revised Manuscript****, where the fluctuating pressure RMS (P*_rms_*) is calculated.*

13. **Comment**: “(Page: 25-32 & 32-36, Line: 388-507 & 508-603)In the manuscript there are too many figures that I think they can be reduced because they have the same meaning. For example, the data in Figs 13-22 are based on the kinetic energy which is based on velocities quantities. Also, the Figs 23-26 are based on vortex structure which are also depend on velocities quantities so the results of increasing or decreasing tends will be the similar for that in Figs. 13-22.”

**Response**: *Thank you for your insightful comment. Fig. 13 presents the flow pattern during t = 4.9s~6.0s under the same conditions. Figs. 14-22 present the distribution and variation of turbulent kinetic energy (k), Figs. 23-26 present the vortex structures identified using Q* criterion. We agree that both k and Q* are derived from the velocity field, and therefore some similarity in their trend may be observed under certain condition. There are some different and complementary purposes between Figs. 14-22 and Figs. 23-26. Figs. 14-22 present the distribution and variation of k, which is a scalar quantity representing turbulence intensity and energy level. There results are directly used to analyze the correlation between turbulence intensity, fluctuating pressure, and underwater noise, which constitutes a key quantitative part of the present research. On the other hand, Figs. 23-26 present the vortex structures, Q* criterion, which describe the instantaneous coherent flow structures and their spatial evolution. These figures are intended to provide a physical interpretation of the energy transfer process and noise generation mechanism, rather than a quantitative measure of turbulence intensity. Although both analyses originate from the velocity field, k and Q* represent energy intensity and flow topology, respectively. The vortex structure visualization cannot be replaced by k distributions, as the latter do not capture the spatial organization and evolution of coherent vortices.*

**Reviewer #2:**

1. **Comment**: “The original intents of the authors are interesting though and they should (a) provide more experimental results, (b) present them in a more understandable way and (c) assess the numerical grid convergence more properly.”

**Response**: *Thank you for your detailed and constructive comment. Regarding the above-mentioned issues, the following responses are given.*

(a) *We agree that the experimental test bench allows for extensive measurements under various hydraulic conditions. In the present study, however, the experimental design intentionally focused on a representative pressure measurement point located 30 cm downstream of the weir, which is nearly coincident with the hydrophone position. The main objective of the experimental campaign was not to characterize the full spatial distribution of pressure fluctuations, but rather to establish a direct and quantitative relationship between local fluctuating pressure and underwater noise generation under different values of Q, H and h. Using a single, well-defined measurement location allowed for consistent comparisons across all tested conditions and with the numerical simulations. Two pressure-based quantities are analyzed: underwater noise sound pressure level (SPL) and fluctuating pressure SPL. These quantities are standard acoustic indicators widely used to characterize respectively the overall noise energy and the intensity of unsteady pressure fluctuations. Nevertheless, we acknowledge that this choice represents a limitation of the experimental dataset. In the revised manuscript, we clarify necessary explanation about this problem* ***(on Page 5, Lines 11-13 and 17-19, and Page 6, Lines 14-17 in the Revised Manuscript )*** *.*
(b) *Considering suggestions for modifying Figs. 6-7, we thank the reviewer for this valuable comment and fully agree that the three-dimensional representations in Figures 6 and 7 suffer from perspective effects, which hinder precise quantitative interpretation. To address this issue without unnecessarily increasing the number of figures in the Manuscript, in the revised* ***three-dimensional figures*** *(****on Page 17, Fig. 6 and Page 18, Fig. 7 in the Revised Manuscript****), the exact numerical values corresponding to each condition are explicitly annotated, allowing the reader to directly identify the precise levels of L_pw_ and fluctuating pressure SPL as functions of H, Q, and h, despite the three-dimensional representation.*

*Considering suggestions about “Pearson correlation factors between L_pw_, SPL, Q, H and h are interesting yet quite expected. The authors should consider determining the R-2 factor to ensure the causality relation between the evolution of the different variables ”, we thank the reviewer for this insight comment. We agree that Pearson correlation coefficients alone mainly reflect the strength of linear association and do not, bu themselves, establish causality. In the present study, the inflow rate (Q), weir height(H), and downstream static water depth (h) are independent control variable, which are prescribed by the experimental design and do not exhibit intrinsic interdependence. Therefore, correlation or regression analysis among Q, H, and h is not intended to infer causal relationships, but rather to quantify their individual influence on the response variables. For the relationships between the control variables (Q, H, and h) and the response variables (underwater noise level L_pw_ and fluctuating pressure SPL), Pearson correlation analysis is employed as a descriptive statistical tool to assess the relative strength and direction of influence, rather than to establish strict causality. By contrast, the relationship between L_pw_ and fluctuating pressure SPL is physically motivated by the underlying flow acoustic coupling mechanism and is central to the objectives of this study. For this reason, in addition to correlation analysis, a linear regression analysis has been performed between L_pw_ and SPL. The corresponding coefficient of determination (R^2^) is now reported in the Revised Manuscript (****on Page 20, Fig. 8)*** *to quantify the goodness of fit and the consistency of this relationship. This regression serves as a quantitative for the subsequent mechanistic analysis of underwater noise generation. Also, we have revised the Manuscript to clarify the distinct roles of correlation analysis and regression analysis, as follows* ***( on Page 18, Lines 8-10 in the Revised Manuscript)****: Pearson correlation analysis is used to quantify the strength of association between variables, while linear regression between L_pw_ and fluctuating pressure SPL is applied to evaluate their quantitative relationship and provide a basis for mechanistic interpretation.*

*Considering suggestions for modifying Figs. 9-11, the FFT-based spectral presented in Figs. 9-11 in the original Manuscript were too small to allow the reader to clearly identify the dominant frequency peaks discussed in the text. To address this issue, we have reprocessed and redrawn the frequency-domain with improved resolution and clearer axis scaling. The full-resolution FFT spectra for all tested operating conditions are provided in the* ***Appendix A.*** *Frequency spectra of underwater noise under all experimental condition* ***(on Pages 56-61, Figs. A-1~A-5 in the Revised Manuscript)****, where the spectral details can be examined without space limitations. In the main text, only representative cases are retained to highlight the key frequency characteristics, while avoiding an excessive number of figures. These modifications substantially improve the readability and transparency of the frequency analysis and provide a more solid experimental basis for the subsequent discussion and numerical comparison.*

**Appendix A.** *Frequency spectra of underwater noise under all experimental conditions*

a) *Q*=15 *L*/s	b) *Q*=30 *L*/s

c) *Q*=45 *L*/s	d) *Q*=60 *L*/s

e) *Q*=75 *L*/s

Fig A-1. Frequency spectra of underwater noise under *H*=50cm

a) *Q*=15 *L*/s	b) *Q*=30 *L*/s

c) *Q*=45 *L*/s	d) *Q*=60 *L*/s

e) *Q*=75 *L*/s

Fig A-2. Frequency spectra of underwater noise under *H*=60cm

a) *Q*=15*L*/s	b) *Q*=30*L*/s

c) *Q*=45*L*/s	d) *Q*=60*L*/s

e) *Q*=75*L*/s

Fig A-3. Frequency spectra of underwater noise under *H*=70cm

a) *Q*=15*L*/s	b) *Q*=30*L*/s

c) *Q*=45*L*/s	d) *Q*=60*L*/s

e) *Q*=75*L*/s

Fig A-4. Frequency spectra of underwater noise under *H*=80cm

a) *Q*=15*L*/s	b) *Q*=30*L*/s

c) *Q*=45*L*/s	d) *Q*=60*L*/s

e) *Q*=75*L*/s

Fig A-5. Frequency spectra of underwater noise under *H*=90cm

(c) *In the revised study, the grid convergence assessment has been reformulated using fluctuating pressure SPL as the primary convergence criterion, rather than only the mean pressure value* ***(on Page 15, Lines 5-12 and Page 16, Fig 5 in the Revised Manuscript)****. This approach is more consistent with the acoustic focus of the paper and directly relates mesh resolution to the quantities analyzed in the core of the manuscript. The added content is as follows: Since underwater noise SPL is derived from pressure fluctuations, the numerical model is further validated by comparing simulated fluctuating pressure with experimental measurements, ensuring that the selected mesh resolution is adequate for acoustic-related analysis. As shown in Fig. 5 b), the shaded gray region corresponds to a ±5% error band based on the experimental measurements. The close agreement between the simulation results and the experimental data, with most simulation points falling within the 5% error band, also demonstrates the reliability and accuracy of the numerical model.*

*Fig 5 b) Comparison of fluctuating pressure*

*We sincerely thank the reviewer for the thorough and insightful evaluation. We fully recognize the validity of the reviewer’s remarks and have undertaken substantial revisions to (a) significantly enhance the experimental dataset and its presentation, (b) improve the clarity and interpretability of the experimental results, and (c) perform a more appropriate numerical grid convergence analysis based on acoustic quantities. We believe that these changes substantially strengthen the scientific rigor of the study.*

We appreciate for Editor/Reviewers’ warm work earnestly, and hope that the correction will meet with approval.

Once again, thank you very much for your comments and suggestions.

Sincerely yours,

Daguo Wang

On-behalf of authors

-------------------------------------------------

Dr. Daguo Wang, Professor

School of Civil Engineering and Geomatics,

Southwest Petroleum University, Chengdu 610039, P.R. China

Email: [dan_wangguo@163.com](mailto:guzhaoln@mail.xjtu.edu.cn)
